# Supplementary material for: Topological, non-topological and instanton droplets driven by spin-transfer torque in materials with perpendicular magnetic anisotropy and Dzyaloshinskii–Moriya Interaction
Source: Sci Rep. 2015 Nov 9;5:16184. doi: 10.1038/srep16184 (PMC4637900; doi:10.1038/srep16184)
Supplement: Supplementary Information [file srep16184-s1.doc]

**Supplementary Information**

**Topological, non-topological and instanton droplets driven by spin-transfer torque in materials with perpendicular magnetic anisotropy and Dzyaloshinskii–Moriya Interaction**

Mario Carpentieri, Riccardo Tomasello, Roberto Zivieri, Giovanni Finocchio

MOVIES DESCRIPTIONS

**MOVIE 1** NTD mode for *D* = 0.0 mJ/m^2^ and *J* = 8.5 × 10^7^ A/cm^2^. The background colors refer to the *z-*component of the magnetization (red positive, white null, blue negative), whereas the arrows colors indicate the *y-*component (blue positive, black null, red negative).

**MOVIE 2** Spatial distribution of the topological density (red +1, blue -1) for the NTD when *D* = 0 mJ/m^2^ and *J* = 8.5 × 10^7^ A/cm^2^.

**MOVIE 3** NTD mode for *D* = 0.25 mJ/m^2^ and *J* = 8.5 × 10^7^ A/cm^2^. The background colors refer to the *z-*component of the magnetization (red positive, white null, blue negative), whereas the arrows colors indicate the *y-*component (blue positive, black null, red negative).

**MOVIE 4** TD mode for *D* = 2.5 mJ/m^2^ and *J* = 8.5 × 10^7^ A/cm^2^. The background colors refer to the *z-*component of the magnetization (red positive, white null, blue negative), whereas the arrows colors indicate the *y-*component (blue positive, black null, red negative).

**MOVIE 5** Spatial distribution of the topological density (red +1, blue -1) for the TD when *D* = 2.5 mJ/m^2^ and *J* = 8.5 × 10^7^ A/cm^2^.

**MOVIE 6** ID dynamics for *D* = 0.75 mJ/m^2^ and *J* = 8.5 × 10^7^ A/cm^2^. The background colors refer to the *z-*component of the magnetization (red positive, white null, blue negative), whereas the arrows colors indicate the *y-*component (blue positive, black null, red negative).

**MOVIE 7** ID dynamics for *D* = 1.00 mJ/m^2^ and *J* = 8.5 × 10^7^ A/cm^2^. The background colors refer to the *z-*component of the magnetization (red positive, white null, blue negative), whereas the arrows colors indicate the *y-*component (blue positive, black null, red negative).

**MOVIE 8** ID dynamics for *D* = 1.25 mJ/m^2^ and *J* = 8.5 × 10^7^ A/cm^2^. The background colors refer to the *z-*component of the magnetization (red positive, white null, blue negative), whereas the arrows colors indicate the *y-*component (blue positive, black null, red negative).

**MOVIE 9** Spatial distribution of the topological density (red +1, blue -1) for the NTD when *D* = 0.0 mJ/m^2^, *J* = 8.5 × 10^7^ A/cm^2^ and *d_c_* = 40 nm.

**MOVIE 10** Spatial distribution of the topological density (red +1, blue -1) for the NTD when *D* = 0.0 mJ/m^2^, *J* = 8.5 × 10^7^ A/cm^2^ and *d_c_* = 100 nm.
